# Parental Influence on Adolescent Gambling: the Role of Communication, Rules, and Social Support

**DOI:** 10.1007/s10899-025-10471-2

**Published:** 2026-02-04

**Authors:** Karin Boson, Mitchell Andersson, Sevtap Gurdal, Emma Claesdotter-Knutsson, Sabina Kapetanovic

**Affiliations:** 1 Department of Behavioral Studies, University West, Trollhättan, Sweden; 2 Department of Psychology, University of Gothenburg, Gothenburg, Sweden; 3 Department of Psychology, University of Inland Norway, Lillehammer, Norway; 4 Faculty of Medicine, Department of Clinical Sciences, Lund University, Lund, Sweden; 5 Region Skåne, Child and Adolescent Psychiatry, Regional Outpatient Care, Lund University Hospital, Lund, Sweden

**Keywords:** Adolescents, Gambling initiation, Gambling problems, Parent-child communication, Parental attitudes, Parental gambling behavior

## Abstract

Adolescent gambling has reached unprecedented levels in recent years, warranting research attention due to its links with other risk behaviors and mental health issues. This study focuses on the risk and protective factors in parent-adolescent relationships affecting gambling behaviors in Swedish adolescents. We investigated the role of parenting practices, parental gambling issues, and parental attitudes toward gambling. Participants included 553 upper secondary school students who completed an online survey about gambling, parental behaviors and attitudes, and parent–child communication. The analytical sample consisted of 57.8% boys with a mean age of 16.8 years (SD = 0.9). Logistic regression models assessed the relationship between these variables, problem gambling, and gambling initiation. Despite legal restrictions against gambling under 18 in Sweden, 49% of adolescents in our sample reported gambling at some point, 25% reported gambling in the past month, and 10% endorsed problem gambling behavior. Our results indicate that parental acceptance and their own gambling behavior are significantly associated with adolescent gambling initiation, suggesting children mimic parental behaviors. Furthermore, parent–child communication and disclosure were associated with both gambling initiation and problems. Enhancing parental awareness is essential to prevent underage gambling initiation and curb future problematic gambling behaviors, given the influential role of parent–child communication, attitudes, and behaviors on adolescents’ gambling habits.

**Supplementary Information:**

The online version contains supplementary material available at 10.1007/s10899-025-10471-2.

## Introduction

Over recent years, adolescent gambling has reached unprecedented levels that warrant research attention. Current prevalence rates on reported gambling in the past year vary across Europe. Reports from Italy indicate that 52% of 15-year-olds had gambled at least once in the past year, while 6.4% gambled regularly (Favieri et al., 2023). In Ireland, 23% of adolescents aged 15–16 had gambled in the past year, with 10% classified as excessive or problem gamblers (Reynolds et al., 2023). In Northern Cyprus, 5.9% of high school students reported gambling for the sake of financial gain over the previous year, surpassing the prevalence of marijuana use (Çavuşoğlu, 2024). In Sweden, where gambling is legally restricted to those aged 18 years or older, national youth reports indicate that 34% of 16–17-year-olds (38% of males and 29% of females) report past year gambling (Public Health Agency of Sweden, 2022). Of these, 8% of the males and 1% of the females exhibit gambling problems (International Classification of Diseases Eleventh Revision ICD-11, 2019), including loss of control, preoccupation, and deceit over gambling (Public Health Agency of Sweden, 2022).

Underage adolescents report various ways of circumventing legal restrictions, and according to a report from the Public Health Agency of Sweden (2022), 20% engaged in some form of online gambling in the past year. A principal route for adolescent gambling is through physical venues, such as gaming machines in bars and certain restaurants. Parental involvement also plays a role: approximately one in three parents report having gambled with their children, for example by buying lottery tickets or betting on sports or horses. Digital avenues further expand access. Underage gamers are increasingly engaging in in-game lotteries and purchasing loot boxes that provide unknown, gambling-like rewards (Miles et al., 2024). Online gambling additionally allows young people to use their parents’ IDs or bank accounts to register on gambling sites (Public Health Agency of Sweden, 2022).

Currently, the most commonly endorsed gambling activity among adolescents is buying lottery tickets (Public Health Agency of Sweden, 2022). However, gambling preferences appear to evolve with age, as males aged 18–19 exhibit a notable rise in online gambling rates relative to their female counterparts and younger peers. They are also more likely to engage in gambling activities of high addictive liability, such as poker and casino games (Public Health Agency of Sweden, 2022). While self-reported surveys provide a robust measure of prevalence, they are still prone to sources of bias. For example, previous studies demonstrate that social desirability may deter individuals from reporting gambling behavior honestly, which may contribute to a systematic underestimation of the true rate in the population (Reynolds et al., 2023). The increase of online gambling and, to some extent, non-regulated access to online gambling sites has likely contributed to the increase of gambling among adolescents. Indeed, due to their still-developing self-regulatory systems and heightened susceptibility to peer pressure (Blakemore & Robbins, 2012), adolescents—especially males—are vulnerable to developing of problematic and addictive behaviors, such as gambling (Chinawa et al., 2023; Stefanovics et al., 2023; Vinberg et al., 2023). Some studies also suggest that young athletes may be more prone to gambling and developing gambling problems (Vinberg et al., 2023).

There are a number of critical psychosocial risk factors for adolescent gambling problems, such as early onset (Sharman et al., 2019), internet addiction (Chinawa et al., 2023), exposure to trauma (Stefanovics et al., 2023), heightened sensation seeking (Hollén et al., 2020), having positive attitudes toward gambling (Hanss et al., 2015), and school disconnectedness (Dickson et al., 2008). Gambling is often linked to other risk behaviors, such as substance use and mental health issues, including depression and suicidality (Stefanovics et al., 2023). In addition, social influences, such as friends and family, have been highlighted as particularly important (Hanss et al., 2015). Still, there is a need for further analysis of adolescent gambling behaviors (Favieri et al., 2023) particularly concerning the risk and protective factors stemming from parent-adolescent relationships.

### Parenting Adolescents and Gambling

Parenting is considered one of the most critical factors pertaining to development of addictive problems in adolescents (Casey et al., 2011; Hanss et al., 2015; Public Health Agency of Sweden, 2022; Yap et al., 2017). Through parenting practices, such as setting rules, monitoring adolescent activities, and fostering supporting attitudes and emotional bonds, parents can help protect their adolescent children from behavioral problems and promote healthy development (Darling & Steinberg, 1993). Indeed, one of the most cited meta-analyses on parenting and adolescent alcohol use (Yap et al., 2017) concludes that parents’ positive attitudes toward adolescent drinking and parents’ own alcohol use are risk factors, while supportive parenting as well as parental knowledge of adolescent whereabouts are important protective factors of adolescent drinking. Given the similar etiology in adolescent risk behaviors such as smoking, drinking, and sexual activities (Duell et al., 2018; Romer et al., 2017), comparable parenting factors may be related to adolescent gambling problems.

Even though gambling is one of several risky behaviors that adolescents engage in today, it is often not perceived as a risk by parents and is sometimes an activity they participate in together (Felsher et al., 2003). In fact, parents may occasionally purchase lottery tickets for their children or gamble on sports events together to win money. Having a liberal attitude towards gambling as a parent can influence adolescents’ own gambling behavior, which has been shown to increase and occasionally develop into problematic gambling over time (Delfabbro & Thrupp, 2003). McComb & Sabiston’s (2010) literature review highlights the multifaceted role of family factors in adolescent gambling, emphasizing that gambling behaviors do not develop in isolation but are embedded within broader family dynamics. Specifically, they suggest that a warm and supportive family climate, along with clear rules and limits regarding gambling, parental monitoring of adolescent activities, and open discussions about risks, serve as protective factors. In contrast, adolescents who grow up in households where gambling is normalized or encouraged, whether through explicit acceptance or parental modeling, are more likely to engage in gambling themselves.

Parental knowledge of adolescents' whereabouts and activities, including knowing what their children are doing and who they are spending time with (Kerr & Stattin, 2000), appears to play an important role in adolescent gambling. Indeed, an Italian study involving nearly 20,000 17-year-olds revealed that adolescents who reported higher levels of parental knowledge not only had lower levels of gambling and gambling problems, but had also more negative attitudes toward gambling and a greater awareness of its harmful effects (Canale et al., 2016). Notwithstanding, parental knowledge also seems to have a long-term effect on gambling, with studies showing that high parental knowledge of adolescent whereabouts and activities in early adolescence (ages 11–14) is linked to less likelihood of gambling problems in young adulthood (age 22) (Lee et al., 2014). Thus, parental engagement in the form of having knowledge of adolescents' general activities and behaviors appears to play a role in adolescent gambling, similar to findings from studies on other risk behaviors and mental health problems in adolescents (e.g., Kapetanovic & Skoog, 2021; Kerr & Stattin, 2000). Given that adolescent disclosure (i.e., openly communicating with parents) is the strongest predictor of parental knowledge (Liu et al., 2020), it is possible that parent-adolescent communication plays a role for adolescent gambling. Previous findings have especially emphasized importance of mother-child communication linked to gambling problems among adults and suggested mother-child communications as an area for prevention (Zhang et al., 2023). The role of parent-child relation (both mother and father) and its potential link to gambling initiation and indication of gambling problems is yet to be understood.

Besides the parent-child relationship, there are several developmental and contextual factors at play during mid-adolescence (i.e., ages 15–17). Individuals younger than 18 are legally minors and not allowed to, for example, drink alcohol, smoke, or gamble. This period is also characterized by growing autonomy and heightened importance of peer influence and group belonging. Compared to 18-year-olds, 15–17-year-olds are more impulsive and engage in more risk-taking behavior (Steinberg et al., 2018), which may shape gambling habits. At age 18, gambling risk increases for different reasons—namely, legal access to gambling, greater autonomy, and independent income (Livazović & Bojčić, 2019). These differing risk profiles highlight the importance of comparing gambling behaviors across developmental stages.

### Current Study and Aim

In this study, we examine the role of parenting practices, parental gambling issues, and parental attitudes toward gambling in relation to Swedish adolescents' gambling behavior. Grounded in the theoretical framework of parental influence on adolescent development (Darling & Steinberg, 1991), we propose two explicit hypotheses and one research question:

First, based on findings from meta-analyses on adolescent alcohol use (Yap et al., 2017), we hypothesize that open parent-child communication and disclosure, strict parental rules, and higher perceived parental social support will be negatively associated with both gambling initiation and problem gambling. Second, as suggested by McComb & Sabiston (2010), we hypothesize that parental gambling and parental acceptance toward adolescent gambling will be positively associated with adolescent gambling initiation and problem gambling. Finally, we explore whether adolescent age possibly moderates the associations between parenting factors and gambling, as age is closely linked to legal access and opportunity to gamble.

## Method

### Procedure

This is a sub-study from a larger investigation on gambling habits and attitudes among athlete and non-athlete high school students (Miles et al., 2025). To recruit participants, we contacted principals from all 20 upper secondary schools in the Skåne region of Sweden that offered both sports-tailored (which included a substantial, though not explicitly defined, component of sport‑specific training integrated within regular school hours) and traditional curricula. Initial contact was made through telephone calls and emails (authors ECK, MJA, SK), where principles were provided written information about the study. Of the 20 contacted, 11 school principals agreed to participate and aid in distributing the survey to students. The survey was then sent to participating schools, either through principals or, in some cases, directly to teachers. The co-authors ECK and SK maintained weekly contact with the responsible personnel at the schools to answer any questions. The online survey was subsequently provided to students during organized mentoring sessions, wherein they were given time to complete it. The survey was available from January 16 to February 17, 2023 and a reminder was sent on January 30, 2023. All responses were anonymous, and participation was entirely voluntary. Participants were required to provide informed consent at the start of the survey to complete it.

### Participants

**Fig. 1:**
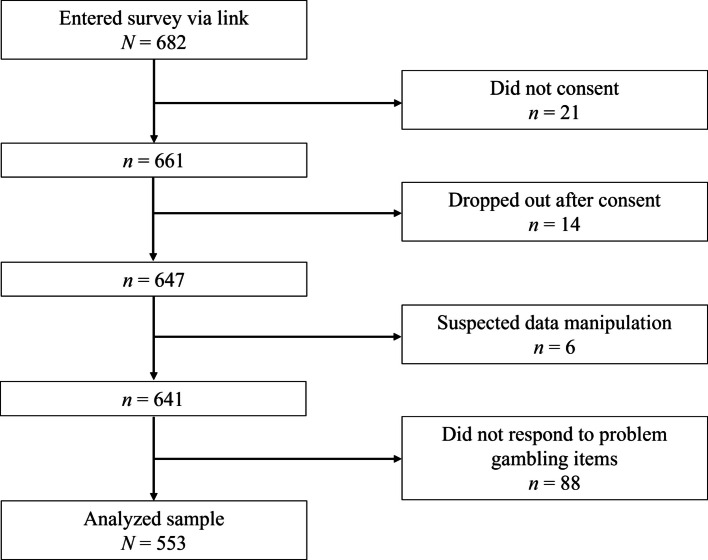
Participant exclusion flowchart. *Note*. Participants removed for suspected data manipulation were flagged due to inconsistent or improper responses across multiple items

Most of the participants were in their first year of upper secondary school (45.4%) and identified as boys (57.8%). Mean age was 16.8 (*SD* = 0.9). All but 5.1% lived with their parents. Additional sample characteristics and descriptions of study variables are displayed in Table 1. Students who did not provide consent, were younger than 15 years of age, refrained from answering any study items, exhibited suspected data manipulation or inaccuracy, or failed to respond to problem gambling-related questions were excluded. We defined data manipulation as aberrant response patterns likely causes by inattention or deliberate falsification. Specifically, this included cases where participants answered “Yes” to all neurodevelopmental disorder (NDD) screening items (e.g., ADHD, ASD, learning disorder) in combination with later non-engagement or uniform response patterns (e.g., responding ‘1’ to all items on subsequent scales). Responses falling outside plausible ranges (e.g., age < 15 years) or internally contradictory (e.g., endorsing problem gambling while denying any gambling activity) were also considered divergent and excluded. Additionally, ten participants who initially endorsed problem gambling behaviors but no lifetime gambling experience, and later indicated that they had never gambled, were excluded. This led to a final analytical sample of 553 participants (see Fig. 1).

**Table 1:** Study sample characteristics

Variable	Missing	Overall (*N* = 553)
*Demographics*		
Age,* n (%*)	0	
18 +		124 (22.4%)
< 18		429 (77.6%)
Class Year, *n* (%)	0	
1st		251 (45.4%)
2nd		205 (37.1%)
3rd		97 (17.5%)
Curriculum, *n* (%)	0	
Sports-Tailored		288 (52.1%)
Traditional		265 (47.9%)
Gender, *n* (%)	5	
Boy		317 (57.8%)
Girl		224 (40.9%)
Other		7 (1.3%)
*Gambling Modalities, n (%)*	0	
Lifetime		
Online casino		90 (16%)
Horse betting		33 (6%)
Store sports betting		19 (3%)
Online sports betting		123 (22%)
Online poker		67 (12%)
Online bingo		70 (13%)
Offline cards/poker		168 (30%)
In-game gambling*		118 (21%)
Past 30 Days	10	
Online casino		41 (7%)
Horse betting		8 (1%)
Store sports betting^†^	4	3 (1%)
Online sports betting^†^	1	61 (11%)
Online poker		22 (4%)
Online bingo^†^	1	25 (5%)
Offline cards/poker^†^	2	65 (12%)
In-game gambling^†^	2	44 (8%)
*Parenting Variables, M* ± *SD*		
Communication/Disclosure (General)	57	41.3 ± 7.0
Parental Rules	66	5.9 ± 1.8
Maternal Social Support	94	29.8 ± 5.3
Paternal Social Support	105	29.4 ± 5.7
Parental Attitudes Toward Child Gambling	20	6.9 ± 3.8
Communication/Disclosure (Gambling)	20	18.5 ± 5.1
Past Year Parental Gambling, *n* (%)	24	173 (32.7%)

Descriptive statistics provided after pairwise deletion. NODS-CLiP = National Opinion Research Center DSM-IV Screen for Gambling Problems. * In-game gambling refers to gambling within video games. ^†^Ten cases were removed due to participants indicating recent gambling but not lifetime gambling for respective modalities

### Measures

#### Demographic Variables and Gambling Modalities

As individuals must be 18 years or older to engage in gambling on regulated platforms, we dichotomized age into two categories: “18 years or older” and “younger than 18”. Gambling type and frequency (lifetime and past 30 days) were assessed. The following gambling types were included: online casino, horse betting, store-based sports betting, online sports betting, online poker, online bingo, offline cards/poker, and in-game gambling (one of the newest forms of gambling, characterized by gambling-like mechanisms embedded within digital games).

#### Problem Gambling

To identify problem gambling, we used the short form of the National Opinion Research Center DSM-IV Screen for Gambling Problems (NODS-CLiP; Toce-Gerstein et al., 2009), a brief screening tool designed to efficiently identify individuals who may have gambling problems. The scale consists of three items assessing core aspects of problematic gambling, asking whether responders had ever lost control over their gambling, lied about their gambling, or reported preoccupation with gambling (Cronbach α =.73). The responses are dichotomous (no = 0 and yes = 1). Answering “Yes” to at least one item suggests a heightened risk for gambling problems.

#### Parent-Child Communication and Disclosure (General)

Parent–child communication and disclosure were assessed using Kerr and Stattin’s (2000) measure assessing parental general knowledge of adolescent activities. The assessment included 12 items examining the extent to which parents inquire about and child disclose/conceal information on various topics, such as school time and post-school activities and online interactions (e.g., “Do your parents know what you do in your spare time?”). Item responses were provided on five-point Likert scales ranging from 1 (“Almost never”, “Not at all”, or “Never”) to 5 (“Very often”, “Very much”, or “Almost Always”). A cumulative score was calculated by taking the sum of each item per participant, with possible scores ranging from 12–60. A higher score indicates open communication and disclosure between the child and their parents. Internal consistency demonstrated satisfactory reliability (Cronbach α =.81).

#### Parental Rules

Using the parental monitoring measure (Conger et al., 1994), we assessed how often parents set rules for their children concerning with whom they socialize, their internet use, their use of money, and what they do after school using five items rated on four-point Likert scales (e.g., “How often do your parents set rules about what you do on the Internet?”). Item responses ranged from 1 (“Never”) to 4 (“Always”). The sum of the four items was used as a composite score, with possible scores ranging from 4–16, with a higher score indicating more stringent rule-setting by parents on their child’s behavior. Internal consistency demonstrated poorer but acceptable reliability (Cronbach α =.60).

#### Maternal and Paternal Support

We assessed maternal and paternal support using the Parent Safe Haven (Biesecker, 2007; Tilton-Weaver et al., 2010). Participants rated their agreement with statements separately for their mother and father, reflecting key aspects of parental support. These statements assessed the perceived availability of maternal and paternal support, encouragement to explore new endeavors, openness to sharing intimate thoughts and emotions, parental comfort during emotional distress, and parental encouragement in pursuing personal aspirations (e.g., “When I am angry, sad or worried, my mom/dad makes me feel better”). Responses were rated on a seven-point Likert scale, ranging from 1 (“Extremely disagree”) to 7 (“Extremely agree”). The cumulative score, derived from the sum of these five items, yielded a possible score range of 5 to 35. Internal consistency demonstrated robust internal consistency for mother support (Cronbach α =.85) and father support (Cronbach α =.87).

#### Perceived Parental Acceptance of Adolescent Gambling

We assessed adolescents’ perceptions of parental attitudes toward gambling using a custom measure consisting of three items. Participants rated the extent to which they believed their parents: (1) found it acceptable for their adolescent child to gamble, (2) held the belief that adolescents should not gamble, and (3) were indifferent to whether their adolescent child gambled or not. Responses were rated on a seven-point Likert scale ranged from 1 (“Extremely disagree”) to 7 (“Extremely agree”). Items were summed to create a total score, with possible scores ranging from 3 to 21. A higher score indicated that adolescents perceived their parents as more accepting of gambling. A principal component analysis supported a one-factor solution (eigenvalue = 2.00), explaining 66.9% of the variance. Factor loadings ranged from.79 to.83, with acceptable sampling adequacy (KMO =.69) and a significant Bartlett’s test of sphericity, χ2(3) = 179.58, *p* <.001. Internal consistency for this scale was satisfactory (Cronbach α =.67).

#### Parent-Child Communication and Disclosure (Gambling Specific)

Parent–child communication/disclosure concerning gambling exclusively was assessed using four items that inquired how often parents ask about child gambling, how often the child tells their parents when they gamble, how often they hide gambling from their parents, and to what degree their parents are aware of how much time they spend gambling (e.g., Do you usually tell your parents when you have gambled?”. Scales were presented on six-point Likert scales and ranged from 1 (“Almost never” or “Not at all”) to 6 (“Very often” or “Extremely”). An additional option, “I do not gamble”, was provided and appeared either on the lowest or highest end of the scale depending on the context of the item. Scores were summed to create a cumulative score ranging from 4–24, with higher scores indicating more open communication and disclosure between child and parent regarding child gambling behavior. A principal component analysis supported a one-factor solution (eigenvalue = 2.06), explaining 51.4% of the variance. Factor loadings ranged from.43 to.85, with acceptable sampling adequacy (KMO =.68) and a significant Bartlett’s test of sphericity, χ2(6) = 56.30, *p* <.001. Internal consistency for this scale was excellent (Cronbach α =.92).

#### Past Year Parental Gambling

Past year parental gambling was assessed with two items asking whether their mother or father, respectively, had gambled over the past 12 months. Four choices were presented to participants, “Yes, often”, “Yes, sometime”, “No, never”, and “Do not know”. The variable was transformed to binary by coding those that responded with “Yes, often” or “Yes, sometimes” as the comparison group (Yes/1) versus those that selected “No, never” or “Do not know” (No/0).

### Statistical Analyses

Descriptive statistics for categorical variables are presented as frequencies and percentages, while continuous measures are presented as means (*M*) with standard deviations (*SD*). Parenting variables of Likert-nature were treated as continuous. Intercorrelations among parenting and child gambling variables were tested using Pearson and point biserial correlations after pairwise deletion for missing data. To account for variable skewness, 95% confidence intervals (CI) were calculated using 1000 resampled bootstrapped samples. To assess the relationship between these variables and problem gambling, as well as gambling initiation, binary logistic regression models were generated. The assumption of normality concerning the outcome's logit in logistic regressions was assessed visually by plotting the predicted log odds of the outcome against the predictor variable. Crude models estimated the relationship between predictor variables and outcome, while adjusted models estimated the relationship while controlling for age and curriculum for problem gambling models and age and gender for gambling initiation models. Interaction terms were introduced when viable to account for possible differences in the effect of independent variables on the outcome at different covariate levels. For regression analyses, missing data were imputed using multiple imputation by chained equations with 20 imputations and then results were pooled using Rubin’s rule (Rubin, 1987). Continuous and binary variables were imputed using linear and logistic regression, respectively. We conducted sensitivity regression analyses without imputation to verify results.

Effect sizes are presented as odds ratios (OR) with 95% confidence intervals (CI). In adherence to the suggested guideline advocating for a minimum of 10 instances of the outcome per independent variable in multiple logistic regression and considering the limited occurrences of problem gambling among exclusively girls (*n* = 4), our adjusted models only included age and curriculum as covariates (Peduzzi et al., 1996). Our exploratory regression analyses modeling gambling initiation included gender as a covariate as there were a greater number of cases of the outcome among girls. We adjusted for multiple testing using the Benjamini & Hochberg (1995) procedure at test level to account for inflated Type I error. R Statistical Software version 4.3.1 was used (R Core Team, 2023).

## Results

### Sample characteristics

Approximately 49.0% of the participants reported engaging in at least one type of gambling (online casino, horse betting, store sports betting, online sports betting, online poker, online bingo, offline cards/poker, or in-game gambling) sometime during their lifetime, and 26.1% had used one of these modalities in the past 30 days. These mechanisms involve players staking real money or in-game currency (often acquired through monetary transactions) to achieve outcomes of uncertain value. Examples include randomized reward systems (e.g., 'loot boxes'), wagering virtual items with market value (e.g., skins betting), and gambling-style mini-games integrated into broader gameplay environments. A total of 56 participants (10.1%) endorsed at least one of the three criteria for the NODS-CLiP scale and thus were identified as lifetime problem gamblers. Overall, offline cards and poker gambling was the most popular gambling modality that adolescents had tried (30%), followed by online sports betting (22%) and in-game gambling (21%). This trend was similar for those who gambled in the past 30 days (see Table 1).

### Parenting Variables Association With Gambling Initiation

To assess which parenting variables were associated with gambling initiation when controlling for age and gender, we generated logistic regression models. Gambling initiation was defined as responding “Yes” to have ever gambled using online casino, horse betting, store sports betting, online sports betting, online poker, online bingo, offline cards/poker, or in-game gambling. In adjusted models, general parent–child communication and disclosure was negatively associated with gambling initiation, OR = 0.97 [95% 0.94,0.99], *p*_*adj*_ =.03, while parental acceptance toward adolescent gambling, OR = 1.12 [95% 1.06–1.18], *p*_*adj*_ <.001, and past year parental gambling, OR = 2.04 [95% CI 1.36–3.05], *p*_*adj*_ =.002, were positively associated with gambling initiation. In addition, the covariate gender (i.e., being female) was also negatively associated with gambling initiation, OR = 0.21 [95% 0.15, 0.31], *p*_*adj*_ <.001. All crude and adjusted relationships are presented in Table 2. For the past year parental gambling models, excluding those who did not know whether their parents gambled in the past year in the non-imputed dataset yielded stronger yet similar results (*OR*_*crude*_ = 2.64 [95% CI 1.80, 3.91], *p* <.001; *OR*_*adj*_ = 2.26 [95% CI 1.49, 3.46], *p* <.001), see Supplement Table S1.

**Table 2:** Pooled Logistic regression model odds ratios for parental variables predicting adolescent gambling initiation

	Crude	Adjusted^a^
Variable	OR [95% CI]	OR [95% CI]
*Parental Predictors*		
Communication/Disclosure (General)	**0.95 [0.93, 0.98]**	**0.97 [0.94, 0.99]**
Parental Rules	0.94 [0.85, 1.04]	0.93 [0.83, 1.04]
Maternal Support	0.97 [0.93, 1.00]	0.96 [0.92, 1.01]
Paternal Support	0.98 [0.95, 1.02]	0.97 [0.94, 1.01]
Parental Acceptance of Adolescent Gambling	**1.15 [1.10, 1.21]**	**1.12 [1.06, 1.18]**
Parental Gambling (Past year, Yes = 1)	**2.44 [1.67, 3.56]**	**2.04 [1.36, 3.05]**
*Covariates*		
Adolescent Age (18 or older = 1)	1.05 [0.71, 1.57]	
Adolescent Gender (Girl = 1)	**0.21[0.15, 0.31]**	

Missing values were imputed using multiple imputation by chained equations. Odds ratios are unstandardized. OR = Odds ratio, ^a^Adjusted for age (18 + vs. < 18 [reference]) and gender (girls vs. boys [reference]) with Benjamini–Hochberg correction. Significant OR:s in bold

### Parenting Variables Association With Problem Gambling

We performed crude and adjusted logistic regressions to ascertain the effects of parenting variables on the likelihood that participants endorsed lifetime problem gambling after controlling for age and curriculum. Summaries of crude and adjusted models are presented in Table 3. We found that general parent–child communication and disclosure, OR_*crude*_ = 0.92 [95% CI 0.87,0.97], *p* =.002, and gambling-specific parent–child communication and disclosure, OR_*crude*_ = 0.83 [95% CI 0.78,0.88], *p* <.001, were associated with lower odds of child problem gambling. Both general, OR_*crude*_ = 0.93 [95% CI 0.88,0.98] *p*_*adj*_ =.02, and gambling-specific parent–child communication and disclosure, OR_*crude*_ = 0.83 [95%CI 0.78,0.88], *p* <.001, remained significant after controlling for age and curriculum. Results were stable after regressions were reran without imputation and with listwise deletion. For the past year parental gambling models, excluding those who did not know whether their parents gambled in the past year in the non-imputed dataset yielded attenuated but similar results (*OR*_*crude*_ = 1.06 [95% CI 0.56, 1.99], *p* =.86; *OR*_*adj*_ = 1.09 [95% CI 0.56, 2.11], *p* =.81), see Supplement Table S2.

**Table 3:** Pooled logistic regression model odds ratios for parental variables predicting adolescent problem gambling

	Crude	Adjusted^a^
Variable	OR [95% CI]	OR [95% CI]
*Parental Predictors*		
Communication/Disclosure (General)	**0.92 [0.87, 0.97]**	**0.93[0.88, 0.98]**
Parental Rules	1.08 [0.90, 1.29]	1.13 [0.93, 1.37]
Maternal Support	0.94 [0.89, 1.00]	0.95 [0.89, 1.01]
Paternal Support	0.96 [0.90, 1.02]	0.96 [0.90, 1.03]
Parental Acceptance of Adolescent Gambling	1.04 [0.97, 1.12]	1.03 [0.96, 1.11]
Parental Gambling (Past year, Yes = 1)	1.21 [0.66, 2.20]	1.27 [0.68, 2.36]
Communication/Disclosure (Gambling Specific)	**0.83 [0.78, 0.88]**	**0.83 [0.78, 0.88]**
*Covariates*		
Adolescent Age (18 or older = 1)	**2.83 [1.49, 5.37]**	
Adolescent Curriculum (Sports-tailored = 1)	0.71 [0.39, 1.29]	

Missing values were imputed using multiple imputation by chained equations. Odds ratios are unstandardized. OR = Odds ratio, ^a^ Adjusted for age (18 + vs. < 18 [reference]) and curriculum (sports-tailored vs. traditional [reference]) with Benjamini–Hochberg correction. Significant OR:s in bold

### Interaction Between Parental Variables and Age

To assess the influence of key predictors across different age groups interaction terms were introduced into our models. However, none of the interactions yielded statistically significant results. Specifically, the interaction involving general parent–child communication and disclosure and age did not yield significant results, OR = 0.99 [95% CI 0.88, 1.10], Wald χ2(1) = 0.227, *p* =.90. Likewise, the interaction focusing on gambling-specific parent–child communication and disclosure and age also did not reach significance, OR = 0.94 [95% CI 0.82, 1.08], Wald χ2(1) = 0.886, *p* =.38.

## Discussion

In this study, we examined the role of parenting practices, parental gambling issues, and parental attitudes toward gambling on adolescents’ gambling behavior. In line with previous research on parental practices and influence on risk behaviors, we hypothesized that open parent–child communication and disclosure, strict parental rules, and higher perceived parental social support would be negatively associated with both gambling initiation and problem gambling (Liu et al., 2020). We also hypothesized that parental gambling and parental acceptance toward adolescent gambling would be positively associated with adolescent gambling initiation and problem gambling (McComb & Sabiston, 2010). Lastly, we investigated whether adolescent age moderated these relationships as the influence of parenting may vary depending on adolescents’ legal and practical access to gambling.

### Key Findings and Parental Influence on Adolescent Gambling

Although gambling is prohibited for individuals under the age of 18 in Sweden, the results revealed that 49% of adolescents have gambled at some point in their lives, up to 26% have gambled in the past month, and 10% are classified as problem gamblers. These figures suggest that many adolescents engage in gambling despite legal restrictions, often outside the oversight of formal systems. In such contexts, parents may be one of the few remaining sources of guidance and regulation, making their involvement and attitudes toward gambling particularly important for prevention and early intervention. Our results indicate that parent-child communication, particularly disclosure, along with parental gambling and parental acceptance toward adolescent gambling, were all associated with gambling initiation. Additionally, gender was linked to gambling initiation. Effective parent-child communication and being female were negatively associated with gambling initiation, whereas parental gambling and acceptance toward gambling were positively associated. Our findings also indicate that parent-child communication, both general information sharing and specific parent-child dialogue about gambling, is associated with gambling problems. Additionally, age was linked to gambling problems. Effective parent-child communication, especially gambling-specific, as well as being over 18, were the strongest risk factors of gambling problems across our models.

However, within the study sample and with the selected variables, the results did not support our hypothesis that strict parental rules would be beneficial in preventing gambling among adolescents. Similarly, higher perceived parental social support did not seem to impact gambling initiation or gambling problems within our sample of adolescents. One possible explanation is that parental strategies in Sweden are generally not characterized by strict rules, but rather a more permissive and supporting parenting style, which has evolved since the late 1950 s (Trifan et al., 2014).

### The Role of Parent-Child Communication and Parental Behavior

These findings are consistent with research highlighting the importance of the parent–child relationship and the influence of parental behaviors and attitudes in shaping adolescents’ risk-taking. As suggested in theory (Kerr & Stattin, 2000), as well as research on other risk behaviors (Kapetanovic & Skoog, 2021; Yap et al., 2017), communication between parents and their children seems to be the key to more healthy adolescent development. Adolescents who openly share information and communicate with their parents create opportunities for parents to offer guidance and set boundaries when necessary. This is true for adolescents in general but is especially important for those showing signs of problem gambling. Indeed, communication is a bilateral process, with both parent and child participating in the dialogue. A child who is at risk of developing gambling problems might even involve their parents to seek help. In this way, parents can play an active role in shaping their children's decision-making, reinforcing norms around healthy behavior, and intervening early when signs of risky behavior, such as gambling, begin to emerge. In contrast, parents who display permissive attitudes toward adolescent risky behaviors, such as drinking alcohol or gambling, may increase the likelihood that their children engage in these behaviors, particularly when such activities are shared between parents and adolescents (Felsher et al., 2003; Yap et al., 2017). This shared involvement can blur boundaries and normalize the behavior, making it more likely for adolescents to view gambling as acceptable or even encouraged. Parents are among the most central role models for their children (Casey et al., 2011; Hanss et al., 2015), and our results indicate that a parent's own gambling behavior can signal a more permissive and positive attitude towards their adolescents’ potential gambling activities. This can be an important pathway leading to negative gambling habits in the future, rather than preventing behavioral problems and promoting healthy development (Darling & Steinberg, 1993).

### Gender and Age Differences in Adolescent Gambling

Our results are also consistent with previous findings, which highlight gambling as a somewhat gendered activity, with boys and young men engaging in gambling to a greater extent than girls and young women (Public Health Agency of Sweden, 2022). Future research is recommended to explore how parent–child communication about gambling, especially child disclosure, relates to boys and whether it needs to be specifically tailored to them. In this study, we also tested whether adolescent age moderated the links between parenting variables and gambling outcomes. Contrary to our assumptions, no moderating effects of age were found in the associations between parenting variables and gambling initiation or problem gambling. This suggests that parental communication, parental gambling attitudes, and parental gambling behavior are similarly important across different age groups, regardless of the age of the adolescents. Nonetheless, some descriptive differences did appear; older adolescents showed a higher prevalence of problem gambling, as indicated by the significant unadjusted association between age and lifetime problem gambling. This is consistent with previous literature suggesting that as adolescents age and become more autonomous, they may engage in more risk behaviors, including gambling (Duell et al., 2018). It is conceivable that older age and increased autonomy, along with greater access to gambling opportunities and reduced parental supervision, contribute to the risk in the older age group. However, since no significant interaction effects were found, parenting factors appear to exert comparable protection or risk effects regardless of whether children are below or above 18 years of age.

### Limitations

There are limitations to this study that need to be highlighted. First, the cross-sectional design precludes any inference of causal relationships between parenting variables and adolescent gambling behaviors. Future longitudinal studies are needed to examine these dynamics over time. Second, since the adolescents themselves reported both their gambling behaviors and perceived parenting variables, their responses may be subject to reporting bias. For instance, social desirability bias may affect responses, and adolescents might not accurately remember details. Although we assured participants of anonymity, some may still have understated their gambling habits or inaccurately reported their perceptions of parental attitudes and behaviors. Thirdly, 94.9% of our sample reported living at home with parents, stepparents, or legal guardians, but we are unable to determine the percentage within this group living with only legal guardians or other adults. This is relevant as our parenting questions only inquired about communication and attitudes with the child’s mother and father. Thus, our findings may not be generalizable to the subpopulation of children living with other legal guardians or adults. Furthermore, the reliability of some scales, particularly the parental rule scale, was low (Cronbach’s α =.60), which implies that results should be interpreted with caution regarding this construct. In addition, even though we adjusted for age and gender and conducted multiple tests, there is still a risk that the findings are influenced by confounding due to unmeasured factors such as socioeconomic status, parental education, and parental mental health. Finally, despite using a well-validated screening tool to measure problem gambling (NODS-CLiP; Toce-Gerstein et al., 2009), which is very brief (three items), it may not fully capture the breadth or seriousness of gambling problems among young people and potentially cause an underestimation of severeness in our sample. Specifically, the odds ratio results indicate only that endorsement of at least one of the three core items (loss of control, lying, or preoccupation) is likely among those with gambling problems, without specifying which particular symptom(s) are most predictive or prevalent. Thus, although the tool is efficient for screening purposes, it provides limited resolution for understanding the nuanced symptomatology of gambling disorder and may risk oversimplifying heterogeneity in presentation.

We acknowledge that our study relies heavily on social and environmental influences in gambling initiation and related problems. Additionally, it is important to recognize that biological and genetic factors may also contribute to an individual’s propensity to initiate gambling. Twin and family studies have demonstrated heritable components of gambling behavior, suggesting that genetic predispositions interact with environmental exposures to shape vulnerability (Rhee & Ellingson, 2023; Warrier et al., 2024). Neurobiological mechanisms may further increase susceptibility by heightening sensitivity to uncertainty and reward (García-Castro et al., 2022). Thus, gambling initiation and maintenance should also consider biological determinants.

## Conclusion

To sum up, our results show that parents’ attitudes and their own gambling behavior are positively associated with adolescent gambling initiation. This highlights that children seem to imitate parental behavior rather than adhere to parental instructions, at least when considering gambling initiation. In addition, parent-child communication and disclosure were associated with both gambling initiation and gambling problems in particular. This underscores the central role of ongoing, open dialogue between parents and their children in both preventing the onset of gambling behaviors and mitigating their escalation into more serious problems. Further studies are warranted, ideally incorporating interviews with parents and adolescents about the function of parent-child communication and relationships linked to gambling behavior.

Though gambling has increased among younger individuals, the results from this study are important for parents and professionals who work with adolescents. Since parental communication, parents’ acceptance towards gambling, and their own gambling behavior seem to influence adolescents’ gambling habits, it is crucial to enhance parental awareness to prevent young people from starting to gamble before reaching legal age and to limit problematic gambling behaviors in the future.

## Supplementary Information

Below is the link to the electronic supplementary material.

Supplementary file1 (DOCX 15 KB)

## Data Availability

The data are not publicly available, but available from the authors upon reasonable request.
