# Supplementary material for: Parental Influence on Adolescent Gambling: the Role of Communication, Rules, and Social Support
Source: J Gambl Stud. 2026 Feb 4;42(1):321–38. doi: 10.1007/s10899-025-10471-2 (PMC13009114; doi:10.1007/s10899-025-10471-2)
Supplement: Supplementary file 1 — Supplementary file1 (DOCX 15 KB) [file 10899_2025_10471_MOESM1_ESM.docx]

Supplement Table S1.

*Logistic Regression Model Odds Ratios for Parental Variables Predicting Adolescent Gambling Initiation (N = 553; Non-Imputed)*

|  | Crude | Adjusted^a^ |
| --- | --- | --- |
| Variable | OR [95% CI] | OR [95% CI] |
| *Parental Predictors* |  |  |
| Communication/Disclosure (General) | 0.95 [0.93, 0.98] | 0.97 [0.94, 0.99] |
| Parental Rules | 0.93 [0.84, 1.03] | 0.90 [0.81, 1.00] |
| Maternal Support | 0.97 [0.93, 1.00] | 0.97 [0.93, 1.01] |
| Paternal Support | 0.98 [0.95, 1.02] | 0.98 [0.94, 1.01] |
| Parental Attitudes (Adolescent Gambling) | 1.15 [1.10, 1.21] | 1.11 [1.06, 1.17] |
| Parental Gambling (Past year, Yes = 1) | 2.45 [1.69, 3.58] | 2.00 [1.34, 3.00] |
| *Covariates* |  |  |
| Adolescent Age (18 or older = 1) | 1.05 [0.71, 1.57] |  |
| Adolescent Gender (Girl = 1) | 0.21 [0.15, 0.31] |  |
| *Note.* Missing values were imputed using multiple imputation by chained equations. Odds ratios are unstandardized. OR = Odds ratio, ^a^Adjusted for age (18+ vs. <18 [reference]) and gender (girls vs. boys [reference]) with Benjamini-Hochberg correction. Significant OR:s in bold. | | |

Supplement Table S2.

*Logistic Regression Model Odds Ratios for Parental Variables Predicting Adolescent Problem Gambling Among those with Gambling History (N = 271; Non-Imputed)*

|  |  |  |  |  |
| --- | --- | --- | --- | --- |
|  | Crude | | Adjusted^a^ |  |
| Variable | OR [95% CI] | | OR [95% CI] |  |
| *Parental Predictors* |  | |  |  |
| Communication/Disclosure (General) | 0.92 [0.87, 0.96] | | 0.92 [0.87, 0.96] |  |
| Parental Rules | 1.06 [0.88, 1.25] | | 1.05 [0.88, 1.25] |  |
| Maternal Support | 0.94 [0.89, 1.00] | | 0.94 [0.89, 1.00] |  |
| Paternal Support | 0.97 [0.91, 1.02] | | 0.97 [0.91, 1.03] |  |
| Parental Attitudes (Adolescent Gambling) | 1.05 [0.97, 1.12] | | 1.05 [0.97, 1.13] |  |
| Parental Gambling (Past year, Yes = 1) | 1.21 [0.66, 2.21] | | 1.26 [0.68, 2.31] |  |
| Communication/Disclosure (Gambling) | 0.83 [0.78, 0.88] | | 0.83 [0.78, 0.87] |  |
| *Covariates* |  | |  |  |
| Adolescent Age (18 or older = 1) | 2.83 [1.49, 5.35] | |  |  |
| Adolescent Curriculum (Sports-tailored = 1) | 1.40 [0.78, 2.55] | |  |  |
| *Note*. Missing values were imputed using multiple imputation by chained equations. Odds ratios are unstandardized. OR = Odds ratio, ^a^ Adjusted for age (18+ vs. <18 [reference]) and curriculum (sports-tailored vs. traditional [reference]) with Benjamini-Hochberg correction. Significant OR:s in bold. | | | | |
